# One Ring Fit to Rule Them All? An Analysis of Avatar Bodies and Customization in Exergames

**DOI:** 10.3389/fpsyg.2021.695258

**Published:** 2021-12-02

**Authors:** Sara Czerwonka, Adrian Alvarez, Victoria McArthur

**Affiliations:** ^1^School of Computer Science, Carleton University, Ottawa, ON, Canada; ^2^School of Journalism and Communication, Faculty of Public Affairs, Carleton University, Ottawa, ON, Canada

**Keywords:** avatars, avatar customization, exergame, inclusive design, ring fit adventure

## Abstract

With the growing popularity of exergames, researchers have noted the importance of presenting players with customizable avatars to encourage the long-term adoption of healthy behaviors offline. However, the “idealized” avatar bodies presented in avatar customization interfaces can represent limited body types and often problematically represent gender as binary. In this paper, we present a systematic analysis of the avatar customization interfaces of six console-based exergames. Results of our analysis indicate that customization options tend to be limited in exergames, especially with regard to body type and gender. Implications for avatar self-priming, self-identification, and healthy behavior adoption are discussed.

## Introduction

**Figure 1:**
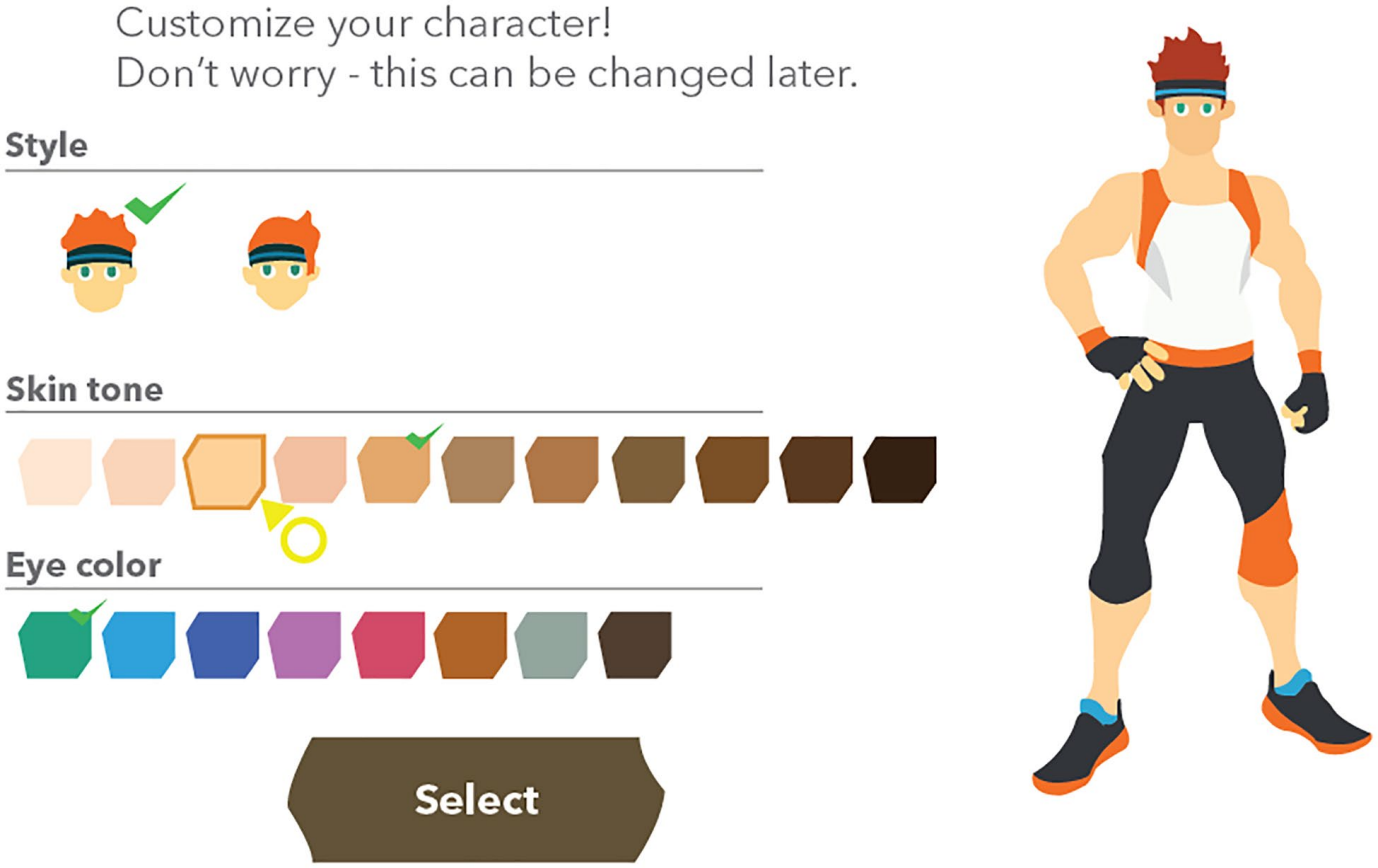
Depiction of the Avatar Customization in *Ring Fit Adventure* (Nintendo Switch).

The growing popularity of exergames in both the public and research spheres has mobilized several game-based exercise interventions on an international scale (e.g., see Ho et al., 2017; Matallaoui et al., 2017). One of the most recent commercial off the shelf exergames, *Ring Fit Adventure* (2019), is an exercise action-role-playing game (RPG) game. Before starting the game, the user creates an avatar that will not only serve as a virtual stand-in for the user, but also a visual feedback mechanism for the exercises performed in-game (see Figure 1). For example, when performing squats, the glutes and legs of the avatar light up. However, if the squats are performed perfectly, these sections of the body light up brightly and appear to be on fire, which indicates that the exercise is being performed most effectively.

Avatar customization options in exergames are typically limited in both quality and quantity, focusing on the customization of athletic gear rather than the avatar’s body. In this paper, we present an analysis of avatar body customizations available in console-based exergames that offer avatar customization, focusing on the choices pertaining to body type and gender. We propose that more options for avatar bodies – choices that not only move beyond the gender-binary but also take different body types into account – would ensure a more socially inclusive design in future exergames involving customizable avatars.

## Related Work

### Avatars

Avatar customization options in games are typically presented to users *via* graphical user interfaces. The quantity and quality of customization options varies greatly from game to game, as does the presentation of these options. Although avatar customization is commonly considered a form of identity expression, researchers have also studied the psychological effects of avatar appearance on user behavior in games and virtual worlds. For example, Yee and Bailenson (2007) proposed the theory of the “Proteus Effect” to characterize the ways in which avatar modification affects how the avatar interacts with others in virtual environments (e.g., users with taller avatars behave more assertively in virtual environments). Interestingly, the authors found that these behaviors can also persist outside of the virtual environment, showing that the transformation is not limited to how users behave in the virtual environment. Their findings are important as they illustrate not only how much effect we have on our avatars, but also how much effect our avatars have on us.

Researchers have also studied the effects of self-priming on avatar self-connection (Jin, 2009, 2010). For example, Jin presents a between-subjects study in which participants created either a realistic Mii or an idealized Mii, studying the effects of self-priming on avatar self-connection (Jin, 2009). Jin noted that participants who created an ideal-self avatar felt a stronger connection to their Mii and their Mii’s in-game achievements. In a subsequent study, Jin studied the effects of realistic and idealized self on users’ perceived interactivity and immersion in *Wii Fit*, an exergame game that uses Miis as player avatars (Jin, 2010). Jin conducted a 2×2 factorial design experiment and noted a greater perceived interactivity among participants who were able to play with an idealized self-avatar (Jin, 2010).

A recent review by Sibilla and Mancini (2018) of 43 studies on user-avatar relationships found that the most frequent types of relationships were *actualization* and *idealization*. They concluded that having an avatar that was closer in appearance to the player correlated with high self-esteem and higher presence and flow and that excessive idealization resulted in negative effects.

In the last decade of user-avatar research, we have noted a departure from the more traditional parasocial perspective in player-avatar relationships in favor of a more social perspective that goes beyond a user-tool connection and recognizes avatar customization as a broader actor-network (e.g., see Banks, 2015; McArthur et al., 2015; Banks and Bowman, 2016; McArthur, 2018; Bowman and Banks, 2020). In each of these works, researchers challenge the user-avatar dichotomy in avatar research and recognize that avatars cannot be studied as a heterogeneous “object,” nor can customized user avatars be studied without understanding the ways in which user interfaces “act” upon their creation as well as the effects of socio-cultural and socio-technical affordances in which they are entangled.

### Exergames

The growing popularity of exergames in both the public and research spheres has mobilized a number of game-based exercise interventions on an international scale (Rosenberg et al., 2010; Song et al., 2011; Staiano and Calvert, 2011; Uzor and Baillie, 2014). Early technologies facilitating console-based exergaming include the EyeToy: Kinetic (Playstation 2, released in 2005) and the Nintendo Wii Remote (2006) and Balance Board (2007). Since then, a number of input devices and peripherals have been developed to support user input in exergames, including the leg strap and Pilates wheel which support gameplay in *RingFit Adventure*.

As with other gaming genres, many console-based exergames offer avatar customization as a feature. In some cases, the avatar being customized serves as a stand-in for the user, and in other cases, the player is customizing their in-game personal trainer. Nintendo’s *Wii Fit* used the user’s own Mii (an avatar tied to their account that could also be used in other games) as their in-game avatar. Although the Mii’s body type can be customized outside of *Wii Fit*, the Mii’s in-game weight was modified when the user stepped onto the Wii Balance Board, a game controller that the users stood on to calculate their weight and track their balance during gameplay. Wii Fit used body mass index (BMI) to determine what the body shape or weight of a player’s Mii should be within the game. Research has shown that BMI is not a very accurate way to track health, as it does not take into account the variability in distribution of lean mass and adipose tissue of individuals (Romero-Corral et al., 2008), meaning that many users who are indeed fit, may be forced to play Wii Fit with an obese-looking avatar due to the fact that lean muscle mass weighs more than fat (Öhman et al., 2014).

A 2018 systematic review in the Journal of Medical Internet Research of 10 randomized trials studying the social effects of exergames on older adults found that “the majority of exergame studies demonstrated promising results for enhanced social well-being, such as reduction of loneliness, increased social connection, and positive attitudes toward others” (Li et al., 2018). Researchers have studied the long-term impacts of exergames on intrinsic motivation and changes in health awareness among participants in longitudinal studies (e.g., see: Brauner et al., 2013; Macvean and Robertson, 2013). According to Bandura (1993), “Self-Efficacy beliefs contribute to motivation in several ways; they determine the goals people set for themselves, how much effort they expend, how long they persevere in the face of difficulties, and their resilience to failures.” Strecher et al. (1986) note that self-efficacy seems to be a predictor of both short- and long-term success in achieving health behavior change.

In addition to self-efficacy beliefs, researchers have also noted the importance of presenting users with fit avatars and avoiding problematic stereotypes relating to body size in encouraging long-term behavioral changes among users (Li et al., 2014; Peña and Kim, 2014). Peña and Kim (2014) report on a study investigating how virtual social cues can be leveraged to influence health behaviors *via* avatar appearance in exergames. The authors explored a combination of player and opponent avatar body types (normal weight vs. obese) and the relationship between these pairings and player exertion in real life. Results of the study indicated that physical activity was boosted when self and opponent avatars had a normal weight, and that it decreased when self or opponent avatars were obese. We take these results to indicate that, as with the Proteus effect described by Yee and Bailenson (2007), which states that the characteristics of an avatar impacts the player’s behavior, the ability to use an idealized avatar in exergames can impact physical exertion and health behaviors in real life.

However, we argue that the notion of an idealized body is personal and will differ from user to user. For example, there are many different types of “fit” bodies (e.g., lean muscle vs. bodybuilders). Furthermore, many avatar customization interfaces notoriously and problematically represent gender as a binary when presenting users with body types in-game (Sundén, 2009). To productively explore the relationship between avatar appearance and offline health behaviors, we propose that first a comparative analysis of avatar customization options in exergames must be conducted.

## Method

We are motivated to study avatar customization in exergames as related work suggests that there is a strong relationship between avatar appearance and the potential long-term changes in health behaviors among players. Considering both Yee’s work on the proteus effect (Yee and Bailenson, 2007), and work of Li et al. (2014) investigating the relationship between fit avatars and long-term healthy behavior adoption, we are interested in a comparative analysis of avatar customization interfaces presented in exergames focusing on customization options relating to the gender and body shape of the avatar. The impetus of this research is to mobilize this analysis in the study of social inclusion in exergame avatar customization interfaces and the effect of social inclusion on player motivation to adopt long-term healthy behavior adoption. This paper presents the first phase of this research: interface analysis. Directions for future work involving longitudinal participant observation are discussed in the final sections of this paper.

### Sample

In this paper, we have opted to focus on console-based games exergames that include avatar customization as a feature. Initially, we had intended to focus on exergames where the player is customizing their own avatar (stand-in) but opted to include any game where players customize an on-screen avatar’s appearance (e.g., games where the on-screen avatar is a personal trainer and not the player’s stand-in) to ensure a larger sample size for analysis. We have excluded games that use a camera capture of the player’s own body (e.g., Shape Up for Kinect) as well as games that fall in the exergame genre but do not include avatar customization features that impact the players’ in-game bodily presence (e.g., Just Dance).

This paper was inspired by the recent release of *Ring Fit Adventure*. In this game, players connect a Joy-Con, one of two one-handed controllers accompanying the Nintendo Switch system, to a Pilates wheel and place the other in a leg strap. The game is a turn-based RPG style in which the player performs movements and battle actions by completing various real-world exercises, such as jogging on the spot, squats, and yoga poses. At the start of the game, the player creates an avatar (see Figure 1) that will serve as their virtual stand-in for gameplay. The player then meets a sentient ring-shaped character (similar in size to the Pilates ring peripheral used to play the game) and they team up to defeat the game’s main antagonist, a bodybuilding dragon named Drageaux.

*Wii Fit*, released in 2007 in Japan and 2008 in North America was an exergame that used a unique platform peripheral called the Wii Balance Board – a device which tracked the user’s center of balance. The game featured yoga, strength training, aerobics, and balance games. The game was considered a breakthrough title in exergaming and spawned more than a decade of exergaming research, including applications in rehabilitation and improving balance in older adults (Nitz et al., 2010).

*Yoga Master* (2019) is an exergame focused entirely on yoga. The game features more than 150 poses that the player can use to build custom 30-min exercise sessions. Player movements can be tracked by the PlayStation Camera or by holding the PS Move controllers, which track real-world movements. The game does not offer player-avatar customization, but rather allows the user to select one of three pre-made avatar trainers named Monica, Sharon, or Asha. Only minor customization options are available for these avatars.

*Kinect Sports Rivals* (2014) was a sports video game developed by Rare and Microsoft Studios for the Xbox One. The game utilizes the Kinect motion-sensing camera to track player movement in several sports activities, including bowling, jet ski racing, target shooting, and tennis. The game uses a combination of traditional avatar customization widgets and facial recognition to support player-avatar creation.

*Fitness Boxing* is an exergame developed by Rocket Company and published by Nintendo for the Nintendo Switch console. Released in 2018 in Japan and 2019 in North America, the game serves as the successor to Gold’s Gym: Cardio Workout for the Nintendo Wii. Players use the Joy-Con controllers to perform boxing moves. Fitness Boxing is another example of an exergame that does not provide a customizable player-avatar but does allow players to customize the in-game trainer’s avatar. The default coach is Lin, but there are five other coaches (three female and two male), and players can customize their skin tone and hair color *via* the avatar customization interface. Players can also unlock further customization options in the form of athletic clothing and accessories *via* gameplay.

*EA Sports Active: Personal Trainer* was a video game developed by EA Canada for the Nintendo Wii Console. The game was released in 2009 in North America and its sequel was released in 2010. Player movement was tracked by holding the Wii Remote in the right hand while using the leg strap to place the Nunchuck controller, a wired one-handed controller that connects to the Wii Remote, on the player’s thigh to track lower body movements. The game featured a series of 20-min workout regimens and a 30-day challenge mode. Players can customize their avatar using the avatar customization interface.

### Method and Methodology

Critical analysis of avatar customization interfaces is largely discursive, providing narrative accounts of limitations on self-representation that are strongly grounded in relevant theories, but fall short at producing productive discourses that contextualize these results in a meaningful way. Recent work by McArthur et al. (2015) presents an analytical framework capable of producing meaningful analyses of the affordances of character creation interface widgets (individual user-interface components), referred to as the Avatar Affordances Framework. The framework is a design ontology that provides a foundation for analyzing the design process as well as designed objects, based on the Function-Behavior-Structure framework proposed by Gero and Kannengiesser (2004):

*Function* describes the purpose(s) for the interface widget (e.g., select hairstyle, etc.).
*Behavior* attributes derivable from the widget (e.g., choose 1 of *n* options, etc.).
*Structure* a technical description of the interface widget (e.g., slider, button, etc.). Additional specifications pertaining to the widget are appended with a colon (e.g., slider: discrete). Where the quantity of choices is derivable from the widget, this number is indicated in round brackets immediately following the structure’s name.

This breakdown allows us to code avatar customization interfaces in a meaningful way and enables direct comparison between games. The Avatar Affordances Framework (McArthur et al., 2015) adds three components to Gero’s framework: Identifier, Hierarchy, and Default.

*Identifier* – what text and/or icons are used to convey the widget’s purpose? (e.g., text: select a gender).
*Hierarchy* – a numerical value indicating a widget’s position in relation to the customization section of a hierarchical interface. For example, a hierarchy value of “2” indicates that the widget is part of a sub-section, while a hierarchy value of “0” indicates that it exists outside the customization section.
*Default* – indicates whether the widget consistently defaults to a particular selection.

By utilizing these six components to categorize different avatar customization interfaces, we can better understand the level of enabled self-representation for different demographics, as well as the interface’s ingrained assumptions about ideal players. This is relevant to the customization options available in exergames, as players with varying body types may want to be represented as a realistic or idealized version of themselves and may be limited by the customization options available. We applied the Avatar Affordances Framework to the character customization interfaces of the selected games, focusing on widgets that modified the avatar’s body shape or physique and gender. This was done by a singular coder, who analyzed video recordings of the avatar customization interfaces of each game to identify the affordances present.

## Results

Using the Avatar Affordances Framework as an analytical method, we were able to break down the interface widgets that allow the player to modify the avatar’s body shape. Ontological data for these widgets are shown in Tables 1, 2. For this analysis, we included any widgets used to adjust the avatar’s physique and/or gender. In modern avatar customization interfaces, players are often limited to the gender binary of male/female, conflating biological sex with gender *via* interface text (Drenten, 2019). This language is relevant in the context of avatar customization, where several interfaces use the term gender when they really offer a binary choice between male and female.

**Table 1:** Avatar affordances data for Gender.

Game	Identifier	Function	Behavior	Structure	Hier.	Default
*RingFit Adventure*	Style	Select sex	Choose 1 of 2	Button(2)	0	Male
*Wii Fit*	Select a gender	Select sex	Choose 1 of 2	Button(2)	0	Player selected
*Yoga Master*	Change coach	Select body	Choose 1 of 3	List(3)	0	Female
*Kinect Sports Rivals*	Now select your Champion’s gender	Select sex	Choose 1 of 2	Button(2)	0	Player selected
*Fitness Boxing*	Change instructor	Select body	Choose 1 of 6	Button(6)	0	Female
*EA Sports Active*	Gender	Select sex	Choose 1 of 2	List(2)	0	Player selected

**Table 2:** Avatar affordances data for Avatar Body.

Game	Identifier	Function	Behavior	Structure	Hier.	Default
*RingFit Adventure*	None	N/A	N/A	N/A	N/A	N/A
*Wii Fit*	Body Test	Modify physique	Modify avatar physique	N/A	N/A	Set by player weight
*Yoga Master*	None	N/A	N/A	N/A	N/A	N/A
*Kinect Sports Rivals*	Physique	Modify physique	Modify avatar physique	Button(2) as discrete slider	0	Set by camera input
*Fitness Boxing*	None	N/A	N/A	N/A	N/A	N/A
*EA Sports Active*	Body Shape	Modify physique	Choose 1 of 4	List(4)	0	“Fit”

Of the six games included in this study, four include a customizable player-avatar and two have a customizable in-game personal trainer. The games that use in-game avatars are *Ring Fit Adventure*, *Wii Fit*, *Kinect Sports Rivals*, and *EA Sports Active: Personal Trainer*. Of these games, *Ring Fit Adventure* is the only game that defaults to a male avatar. The other three games allow the player to make the selection before populating the avatar customization interface. In *Ring Fit Adventure*, players can change the sex of their avatar (presented as “style”), skin tone, and eye color. Other features, such as hair style, hair color, and body shape are pre-set and cannot be changed. All six of the games present customization options related to gender and body type at the top hierarchy level, therefore ensuring that certain customization options are not hidden unintentionally.

Unfortunately, all of the games included in this study conflate sex and gender, offering a binary choice for avatar bodies. The one exception to this is *Yoga Master*, which only offers female trainer avatars. *Wii Fit*, *Kinect Sports Rivals*, and *EA Sports Active* are the only games in this sample that allow users to change the physique of avatar bodies. *Kinect Sports Rivals* presents a slider that allows the user to discretely adjust the physique of the avatar between an idealized fit body and an overweight body. *EA Sports Active* presents the user with a list that allows them to select one of four pre-configured bodies in a similar range. The other three games present the user with a default avatar physique that matches the ideology of the type of exercise offered in-game. For example, *Ring Fit Adventure* presents the user with a body-builder type physique and the avatar bodies in *Yoga Master* are all very slender, stereotypically feminine bodies similar to those that are commonly featured as idealized yogi bodies on Instagram (Hinz et al., 2021).

The structure of the avatar affordances across all six games takes the form of either buttons or lists, except in the case of *Wii Fit*, which uses the player’s own weight to set the avatar body. Additionally, *Kinect Sports Rivals* uses a discrete slider to adjust avatar physique. Both *Kinect Sports Rivals* and *Wii Fit* take advantage of atypical input devices when setting the user’s body shape. Although both games present the user with customization interfaces to fine tune the physical appearance of their avatar, *Kinect Sports Rivals* sets the default physique of the avatar based on images captured by the console’s camera and *Wii Fit* uses BMI measurements generated by the Balance Board.

The way that the *Wii Fit* calculates BMI has been critiqued by researchers. Öhman et al. (2014) note that the game is designed with a BMI of 22 as an ideal body. During player weigh-in, Miis jump up and down with anticipation of their BMI. If the player scores an ideal BMI or lower, the avatar reacts in a positive manner. If the player scores a BMI above the idealized 22, the Mii appears to be sad or embarrassed. In this way, the authors suggest that the game uses shame as a major strategy, even though BMI is known to be a problematic way to determine whether an individual is at a healthy weight (Romero-Corral et al., 2008).

Compared to other genres that offer avatar customization, the number of customizations available in these games was considerably lower (e.g., see McArthur et al., 2015). Customization options offered *via* these interfaces were largely geared toward fitness attire and accessories, rather than body shape. The exception to this is *Ring Fit Adventure*, where upgraded attire provides stat bonuses. Otherwise, most of the additional attire options available in the other games offered aesthetic appeal only.

**Figure 2:**
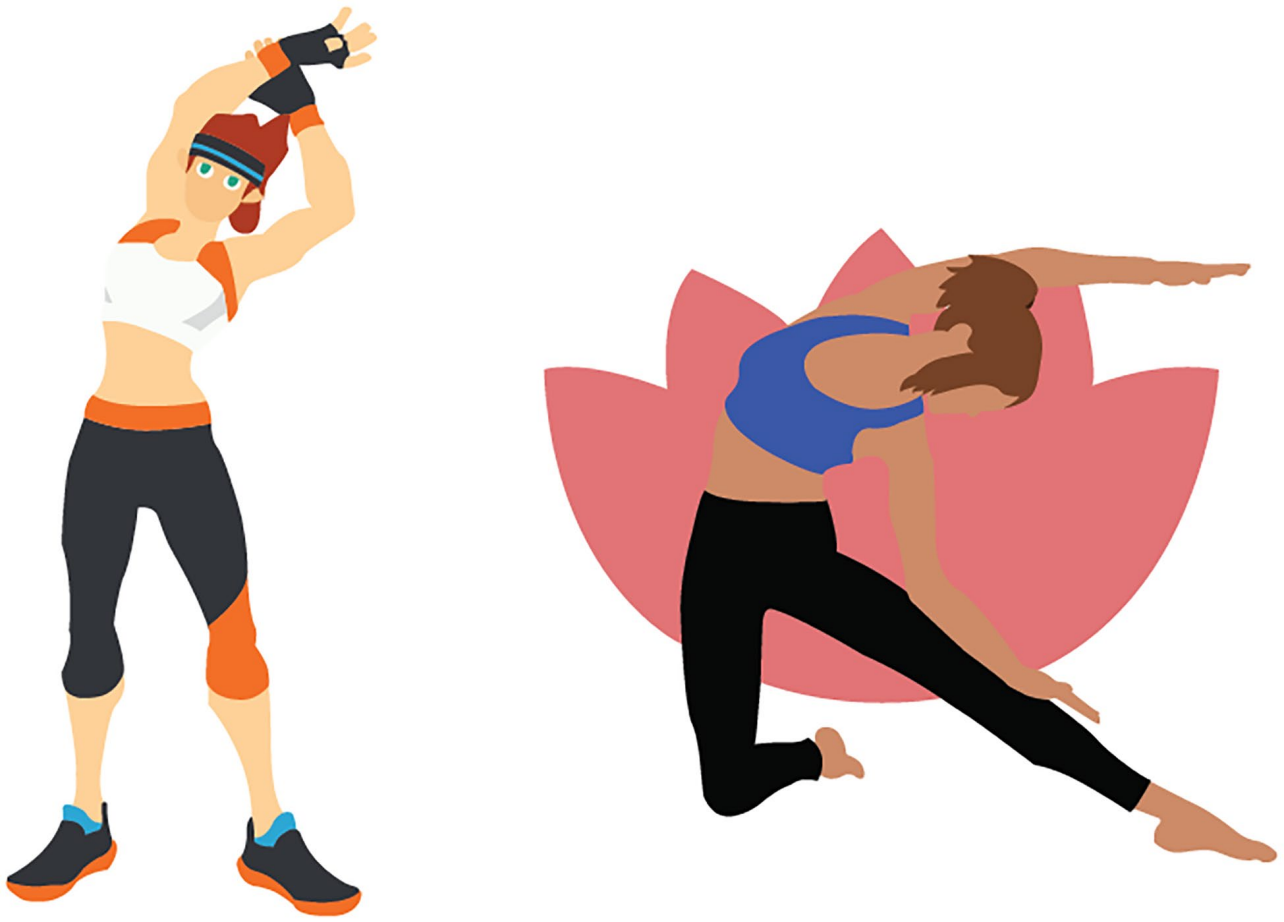
Depiction comparing the female avatar bodies available in *Ring Fit Adventure* and *Yoga Master*.

It is unfortunate that our findings did not reveal more variety in body types available given that there are many different types of bodies that are accepted as healthy. Although the aforementioned research by Peña and Kim (2014) found that being able to choose an idealized body helps to motivate users in exergames, we argue that a broader definition of “idealized” avatar bodies in exergames should be considered by industry to match the more body-positive messaging that is becoming more prevalent in real life. For instance, a visual comparison of the female avatar bodies available in *Ring Fit Adventure* and *Yoga Master* represent two drastically different “fit” or “idealized” women’s bodies (see Figure 2). Thus, we argue that greater inclusion must be built into avatar customization interfaces (e.g., the ability to customize avatar body shapes) and that more nuanced research into motivation in exergames and “idealized” body times is needed. Most importantly, this research must consider that body types are not a binary (e.g., fit vs. obese) and that people who engage in exercise do not all have the same body composition and health goals.

## Discussion

As we have shown in this study, exergames provide users with the ability to create their own avatar – an opportunity for players to try on new identities, or to visually place themselves at the center of an interactive digital adventure. Within this context, the issue of social exclusion arises when players who want to recreate themselves *via* their avatars are limited by interface affordances. As we found in our study, the majority of exergames sampled did not provide an adequately customizable avatar with inclusive options for gender, body type, etc. When this happens, games go from being places where we can be who we want to be, to becoming places where we can only be who we are allowed to be.

The study of affordances is important, as it not only makes visible the ways in which these interfaces may be socially exclusive, but also provides an opportunity to systematically study industry practice and to propose guidelines to help developers design character creation interfaces with social inclusion in mind. This is especially important when considering the advantages of allowing users to create idealized avatars in exergames. We urge industry practitioners to consider a more inclusive model of avatar physique when developing exergames that offer avatar customization.

Although the analysis in this paper offers a point of critique in the study of exergame avatar customization interfaces, we are especially interested in the measured impact of these interface options on long-term player motivation and subsequent healthy behavior changes made in the real world. Additionally, advancements in input technologies present many interesting possibilities for inclusive design in exergaming. We hope that avatar customization interfaces in exergames enjoy similar advancements, working toward socially inclusive avatars in the genre.

## Limitations and Future Work

We recognize that our study has limitations that may impact the results and therefore our perception of avatar customization in exergames. Due to our inclusion criteria, our sample size is much smaller than similar studies of other genres would be (e.g., RPGs). In our future work, we intend to connect our results longitudinal studies with participants observing how such interfaces impact their long-term behaviors, self-efficacy, and motivation.

## Data Availability Statement

The original contributions presented in the study are included in the article/supplementary material; further inquiries can be directed to the corresponding author.

## Author Contributions

SC, AA, and VM contributed to the writing and revision of the article. VM contributed to the design of the work and the methodology. All authors contributed to the article and approved the submitted version.

## Conflict of Interest

The authors declare that the research was conducted in the absence of any commercial or financial relationships that could be construed as a potential conflict of interest.

## Publisher’s Note

All claims expressed in this article are solely those of the authors and do not necessarily represent those of their affiliated organizations, or those of the publisher, the editors and the reviewers. Any product that may be evaluated in this article, or claim that may be made by its manufacturer, is not guaranteed or endorsed by the publisher.
